# Glucose Control in Post-hemolytic-Uremic Syndrome Diabetes: A New Approach Offered by Sensor-Augmented Pump Therapy

**DOI:** 10.3389/fped.2022.882319

**Published:** 2022-05-30

**Authors:** Valeria Grancini, Federica Alessandra Vianello, Santo Colosimo, Alessia Gaglio, Veronica Resi, Maura Arosio, Gianluigi Ardissino, Giovanni Montini, Emanuela Orsi

**Affiliations:** ^1^Endocrinology Unit, Fondazione IRCCS Ca’ Granda – Ospedale Maggiore Policlinico, Milan, Italy; ^2^Pediatric Unit, Fondazione IRCCS Ca’ Granda - Ospedale Maggiore Policlinico, Milan, Italy; ^3^Oxford Centre for Diabetes, Endocrinology and Metabolism, University of Oxford, Oxford, United Kingdom; ^4^Department of Clinical Sciences and Community Health, University of Milan, Milan, Italy

**Keywords:** hemolytic-uremic syndrome, diabetes, insulin pump therapy, continuous glucose monitoring, SAP therapy, insulin

## Abstract

We report the case of a 3-year-old girl admitted to her town emergency department for fever (39°C) associated with diarrhea, generalized edema, oliguria, and drowsiness. The blood test revealed metabolic acidosis, leucocytosis, increased inflammatory markers, anemia, thrombocytopenia, and acute kidney failure. Based on the diagnosis of hemolytic-uremic syndrome, the patient was referred to a third-level children hospital. Assisted ventilation, hemodialysis, and parenteral nutrition were instituted. The blood glucose levels increased above 200 mg/dl with peaks at 500 mg/dl. Islet auto-antibodies were negative and C-peptide was undetectable, thus ruling out the diagnosis of type 1 diabetes. Multiple-daily-injection insulin therapy was then instituted with the following regimen: Detemir 2 U once daily and Aspart 0.5 U if blood glucose >200 mg/dl. Despite the very low insulin dosage, the patient experienced frequent and severe hypoglycemic events during the following 24 h and was therefore switched to sensor-augmented pump therapy. Optimal glucose control was achieved without further hypoglycemic episodes. Moreover, thanks to the possibility to customize insulin therapy hour by hour during the day and the use of a pre-low glucose suspend system, glucose control was maintained even despite the continuous modifications in the nutritional scheme due to the multiple complications that arose during hospitalization. This rare case of post-hemolytic-uremic syndrome diabetes, treated with sensor-augmented therapy from its outbreak, suggests for the first time the potential of this therapeutic strategy in achieving glucose control without significant hypoglycemic episodes in children with secondary forms of diabetes associated with very low insulin requirement.

## Background

Hemolytic uremic syndrome (HUS) is a thrombotic microangiopathy (TMA) affecting predominantly the kidney and characterized by a triad of thrombocytopenia, mechanical hemolytic anemia, and acute kidney injury (1). It affects approximately 0.6 in 100,000 children a year in Europe, with a peak incidence in children aged under 5 years (2). The definition includes several disorders and the most common etiopathological factor is Shiga toxin-producing *Escherichia coli* (STEC) infection (typical HUS). Additionally, HUS can occur as a result of inherited or acquired dysregulation of the alternative complement cascade (atypical HUS or aHUS) and in the setting of invasive pneumococcal infection (2). TMA results in microthrombi formation in the small vessels of several organs, most commonly the kidneys, bowel, central nervous system, pancreas, heart, and liver. Therefore, extra renal manifestations of the disease include also seizures, stroke, bowel ischemia, elevated transaminases, myocardial infarction, hypertension, and diabetes mellitus (DM).

**FIGURE 1:**
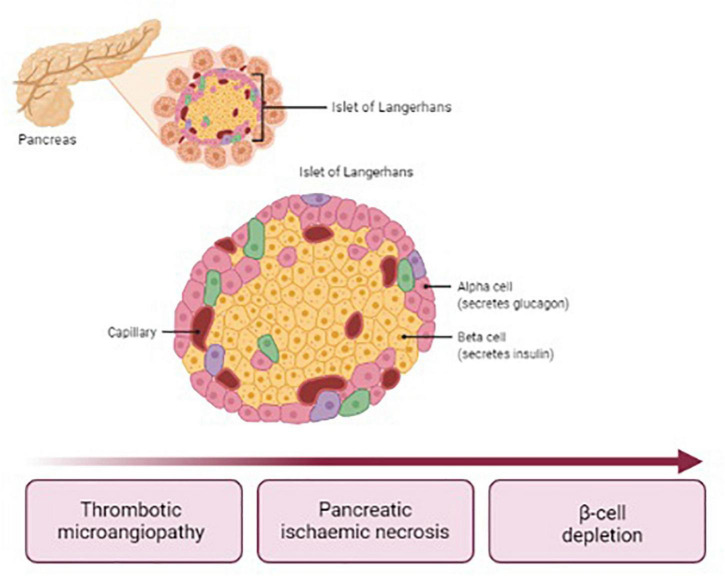
Pathogenesis of post-HUS DM.

Post-HUS DM affects up to 8% of children with STEC-HUS (3, 4). DM usually occurs within 14 days after the onset of HUS symptoms, though later occurrence has been reported (5–7). Data from literature show that HUS complicated with DM is associated with a 23% increase in the case-fatality rate (8). The pathogenesis of post-HUS DM differs from type 1 DM (T1DM), since islets of Langerhans are affected by ischemic damage due to TMA, as illustrated in Figure 1. Therefore, typical auto-antibodies for T1DM are undetectable. Necrosis may affect selectively the endocrine or involve also exocrine pancreas. In any case, both the alpha and beta cells are depleted. The only therapy currently available for post-HUS DM is insulin, usually administered as a basal-bolus scheme (2). The lack of glucagon secretion facilitates the occurrence of severe hypoglycemia whenever insulin bolus does not match the carbohydrate intake. For this reason, and considering that HUS population consists mainly of young children with extremely high insulin sensitivity, the management of blood glucose (BG) control is challenging in all settings, including intensive care unit, in the ward, and at home. The sensor-augmented pump (SAP) is actually considered the gold standard therapy for T1DM and its flexibility and adaptability suit to individuals with minimal insulin requirements. For this reason, such a therapeutic strategy could be particularly effective also in patients suffering from post-HUS diabetes. However, actually there is no evidence demonstrating the efficacy and safety of SAP therapy in these patients. To our knowledge, this is the first report demonstrating the feasibility of such a therapeutic approach in this population.

## Case Report

We report the case of a 3-year-old girl admitted to her town emergency department on 12 November 2019 for fever (39°C) associated with diarrhea, generalized edema, oliguria, and drowsiness. Blood testing revealed metabolic acidosis, leucocytosis, increased inflammatory markers, anemia, thrombocytopenia, and acute kidney failure. Suspecting a HUS, the patient was transferred to the pediatric emergency room of the Fondazione IRCCS Ca’ Granda—Ospedale Maggiore Policlinico. Despite the broad-spectrum antibiotic therapy and supportive therapy, general conditions deteriorated and arterial hypertension ensued during the next days. The total body CT documented the presence of bilateral pneumonia, bilateral pleural and abdominal effusion, ischemia of the kidneys and pancreas, edema of the colon wall and the submucosa of the stomach and duodenum, and massive ileal distension. The search for Shiga toxin genes in the stools was positive, which led to a diagnosis of STEC-HUS.

The bilateral pleural drainage was positioned and hydration and total parenteral nutrition (TPN- considering a recommended caloric intake of 75 kcal/kg/day, according to WHO guidelines) (9) were instituted, together with anti-hypertensive and antiviral therapy for cytomegalovirus (CMV)-DNA positivity. Subsequently, the patient underwent oro-tracheal intubation and invasive ventilation due to an episode of hemoptysis and subsequent acute respiratory insufficiency, as well as hemodialysis and blood transfusions, because of a worsening of renal function, and anemia.

The following days, the BG levels increased above 11 mmol/L with peaks at 28 mmol/L during parenteral nutrition. Glutamic acid decarboxylase and islet cell antibodies were negative and C-peptide was undetectable. Therefore, the following multiple daily injection (MDI) insulin regimen was instituted: Detemir 2 U once daily and Aspart 0.5 U if BG >11 mmol/L. Despite the extremely low insulin dosage, the patient experienced frequent, and severe hypoglycemic episodes during the following 24 h (lowest level of glycemia detected: 1.5 mmol/L), treated with supplementation of oral glucose, infusion of 5% glucose solution, and administration of 33% glucose boluses in case of BG < 3.8 mmol/L. For this reason, MDI was promptly replaced with the SAP therapy (Medtronic MiniMed 640 and Guardian sensor ^®^) set at 0.025 U/h basal rate. An optimal BG control was achieved without the occurrence of hypoglycemia.

During the following weeks, the general conditions and the renal and pulmonary function gradually improved, so that dialysis and assisted ventilation were suspended, drainages were removed, and antibiotic therapy was withdrawn. During the hospitalization, the patient lost 10 kg of body weight due to severe caloric malnutrition. In addition to this, the long bed rest caused weakness, general hypotony, and muscle atrophy. Oral feeding was restarted with additional overnight parenteral nutritional (OPN) in order to gain adequate protein and caloric daily intake (considering a daily caloric requirement of 58 kcal/kg/day). To prevent proximal intestinal malabsorption, pancreatic enzymes were supplemented. Following to dietary changes, the basal insulin infusion rate was modified to 0.015 U/h overnight and 0.05–0.1 U per meal according to carbohydrate (CHO) content (at least 50 g per meal).

After 4 weeks, relapses of intestinal subocclusion occurred and were treated with prokinetics, laxatives, and evacuative enemas. During the acute phase, OPN alternating with minimal enteral feeding with liquid diet (with Alfarè ^®^ Milk or Modulen ^®^ Milk) through nasogastric tube or, in case of re-obstruction, TPN was instituted, with return to *ad libitum* oral dietary intake at the time of remission. Accordingly, two different profiles of basal insulin infusion rate were set, with square wave insulin boluses lasting 3 h when needed. Barium enema showed no intestinal stenosis suggesting a paralytic ileus possibly due massive ischemia, aggravated by severe exocrine pancreatic insufficiency and prolonged bed rest. However, after 2 months of recurring episodes of subocclusion, a new barium enema was performed, showing stricture of the transverse colon that was treated with splenic flexure resection and colo-colic anastomosis. According to the ERAS guidelines (10), early oral feeding alternating with OPN was initiated after the surgery.

The assessment of nutritional status showed improvements in all items, including caloric intake, weight gain, limb waists, muscle strength, and physical performance. After the parents attended a CHO counting training course, an insulin/CHO ratio of 0.1/100 at each meal and a correction factor of 0.1/250 were recalculated. In Table 1 are reported the different insulin schemes according to different nutritional strategies.

**TABLE 1:** Different insulin schemes according to different nutritional strategies.

Nutritional strategy	Insulin scheme
TPN	Insulin basal rate: 00.00–06.00: 0.025 U/h 06.00–18.00: 0.050 U/h 18.00–24.00: 0.025 U/h
OPN + liquid diet	Insulin basal rate: 00.00–06.00: 0.15 U/h 06.00–18.00: 0.050 U/h 18.00–24.00: 0.15 U/h Standard insulin bolus at meals (0.1 U for meals with at least 50 gr CHO)
OPN + minimal enteral feeding *via* naso-gastric tube	Insulin basal rate: 00.00–06.00: 0.025 U/h 06.00–18.00: 0.050 U/h 18.00–24.00: 0.025 U/h 3-h square insulin bolus for enteral nutrition (0.025 U for 8 gr CHO)
OPN + *ad libitum* oral diet	Insulin basal rate: 00.00–06.00: 0.15 U/h 06.00–20.00: 0.050 U/h 20.00–24.00: 0.15 U/h Wizard bolus at meals (Insulin/CHO ratio 0.1/100 at meals. correction factor 0.1/250)

*TPN, Total Parenteral Nutrition; U, Units; OPN, Overnight Parenteral Nutrition; CHO, Carbohydrates.*

**FIGURE 2:**
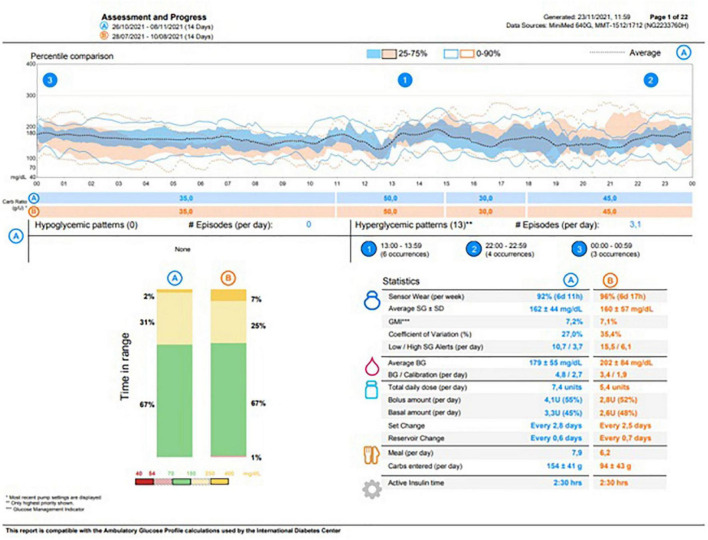
Ambulatory glucose profile at patient’s most recent metabolic assessment.

The child was discharged on 21 February 2020 in good general conditions with persistent mild anemia, hypertension treated with amlodipine, exocrine pancreas failure, and DM. At the moment of discharge, the patient’s ambulatory glucose profile (AGP) showed no hypoglycemic events in the last 14 days. The parents were fully trained about hypoglycemic episode management at home with simple carbohydrates administration (10 g, considering the patient’s body weight) or, in case of severe hypoglycemia (involving loss of consciousness), with intramuscular glucagon injection (0.5 mg). At home, BG control was very well managed through SAP therapy). At the AGP re-evaluation, 14 days after discharge, no severe hypoglycemic events were reported and the episodes of mild hypoglycemia were adequately managed by the parents. Thereafter, the patient was addressed to a local diabetologic center for further follow-up on diabetes. The most recent testing (8 November 2021) revealed a HbA1c of 57 mmol/mol, whereas the AGP showed a glucose management indicator of 7.2% and a time in range of 67% with no hypoglycemic episodes, as reported in Figure 2.

## Discussion

Sensor-augmented pump therapy is a complex multidevice insulin delivery system that operates on the base of preset parameters to help patients with DM and their caregivers in the management of BG control and prevention of hypoglycemia. Briefly, the insulin pump receives information on BG concentration from a subcutaneous sensor that indicates meal and correction insulin boluses and stops insulin delivery in case of a lowering BG trend in order to prevent hypoglycemia. A powerful, solid, and stable software is required to allow smooth communication between the pump and the sensor. In addition, a user-friendly graphic interface provides the patient with real-time control of glycemic trends.

The use of this system for insulin delivery in T1DM and other insulin-dependent types of DM provides several advantages in the management of BG control, especially in patients who require very small amount of insulin and are very sensitive to small increases in insulin infusion. This was the case of our patient with HUS who suffers from an insidious form of DM characterized by wide BG excursions and high insulin sensitivity. The depletion of glucagon secreting alpha-cells of endocrine pancreas provides further complexity for the lack of the counteracting mechanisms for hypoglycemia prevention.

In conclusion, this case report highlighted for the first time how SAP therapy could be a valuable treatment option in both outpatient and inpatient settings when MDI is associated with an increased risk of severe hypoglycemia. While the use of this system for the daily management of BG control is now spreading among patients with T1DM, there are no data from the literature about the use of SAP therapy in inpatient settings and, in particular, about its use from the very early onset of specific critical conditions as post-HUS diabetes.

The potential clinical implications of this findings concern the possibility to resort to this therapeutic strategy whenever glycometabolic conditions require the use of a highly adaptable system of insulin delivery.

The availability and cost of the device and nurse staff education are among the main obstacles to the implementation of this system in the inpatient clinical practice. Moreover, the patients’ and caregivers’ education, especially focussing on carb estimating, is mandatory to reduce the risk of misuse and provide a safe and effective BG control.

## Data Availability Statement

The raw data supporting the conclusions of this article will be made available by the authors, without undue reservation.

## Ethics Statement

Ethical review and approval was not required for the study on human participants in accordance with the local legislation and institutional requirements. Written informed consent to participate in this study was provided by the participants’ legal guardian/next of kin.

## Author Contributions

VG and FV conceptualized and designed the study, drafted the initial manuscript and reviewed, and revised the manuscript. AG and SC collected the data, carried out the initial analyses and reviewed, and revised the manuscript. GA, EO, MA, and GM designed the data collection instruments, coordinated and supervised the data collection, and critically reviewed the manuscript. All authors approved the final manuscript as submitted and agreed to be accountable for all aspects of the work.

## Conflict of Interest

The authors declare that the research was conducted in the absence of any commercial or financial relationships that could be construed as a potential conflict of interest.

## Publisher’s Note

All claims expressed in this article are solely those of the authors and do not necessarily represent those of their affiliated organizations, or those of the publisher, the editors and the reviewers. Any product that may be evaluated in this article, or claim that may be made by its manufacturer, is not guaranteed or endorsed by the publisher.

## Abbreviations

SAP: sensor-augmented pump
HUS: hemolytic-uremic syndrome
BG: blood glucose
MDI: multiple daily injections
TMA: thrombotic microangiopathy
STEC: Shiga toxin-producing *Escherichia coli*
DM: diabetes mellitus
T1DM: type 1 diabetes mellitus
U: units
TPN: total parenteral nutrition
OPN: overnight parenteral nutritional
CHO: carbohydrates.
